# Solid Serous Adenoma of the Pancreas: A Case Report and Review of the Literature

**DOI:** 10.1155/2016/3730249

**Published:** 2016-07-25

**Authors:** Anastasios Katsourakis, Ioannis Dimitriou, Georgios Noussios, Iosiph Chatzis, Efthimios Chatzitheoclitos

**Affiliations:** ^1^Department of Surgery, “Agios Dimitrios” General Hospital of Thessaloniki, 54634 Thessaloniki, Greece; ^2^Laboratory of Anatomy, Department of Physical Education and Sports Medicine (Serres), Aristotle University of Thessaloniki, 56430 Thessaloniki, Greece; ^3^Diavalkaniko Medical Centre, Thessaloniki, Greece

## Abstract

Herein, we report a case of a solid-type serous cystadenoma of the pancreas which is the 16th case reported worldwide and the first ever reported in Greece. Magnetic resonance imaging showed a hypervascular mass in the tail of the pancreas of a 72-year-old female who presented with mild abdominal pain. Distal pancreatectomy was performed by laparotomy and histological and immunohistochemical examination revealed a solid-type serous cystadenoma of the pancreas. Preoperative diagnosis of a solid-type serous cystadenoma of the pancreas is difficult, and, due to its benign nature, simple excision of the tumor is the recommended treatment.

## 1. Introduction

Pancreatic neoplasms, especially adenocarcinomas of the pancreas, are a very serious medical condition and usually demand difficult surgeries that are accompanied by significant mortality and morbidity. In contrast, cystic neoplasms of the pancreas are very rare (only 1-2% of all exocrine pancreatic tumors) [1–3] and, due to their clinical and biological characteristics, form a distinct type of pancreatic tumor that can be treated adequately with simple excision and with very good prognosis [1–4]. Cystic neoplasms are mainly divided into two categories: serous and mucinous. Serous cystic neoplasms of the pancreas are subdivided histologically into five types: serous microcystic adenoma, serous oligocystic ill-demarcated adenoma, solid serous adenoma, von Hippel-Lindau-associated cystic neoplasm, and serous cystadenocarcinoma [1, 2, 4–7]. Despite their histological similarity, these five subtypes of serous cystadenoma have different characteristics regarding size, location, and biology, and the solid serous cystadenoma (SSC) is the rarest of the five subtypes [1, 2, 4–7].

## 2. Case Presentation

**Figure 1:**
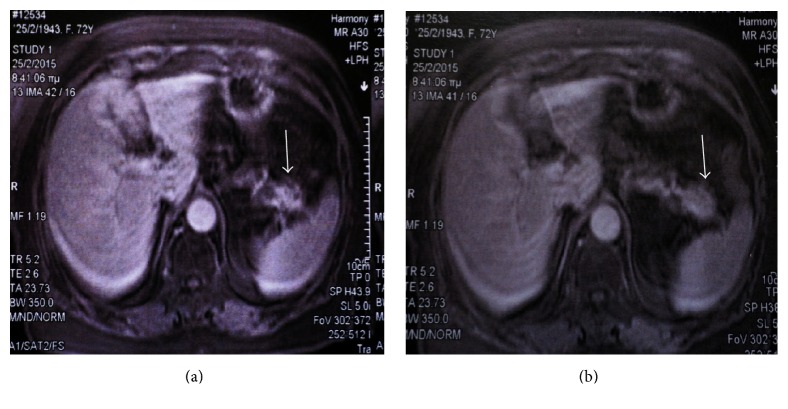
A mass in the tail of the pancreas with (a) strong enhancement and with (b) rapid washout, MRI images.

**Figure 2:**
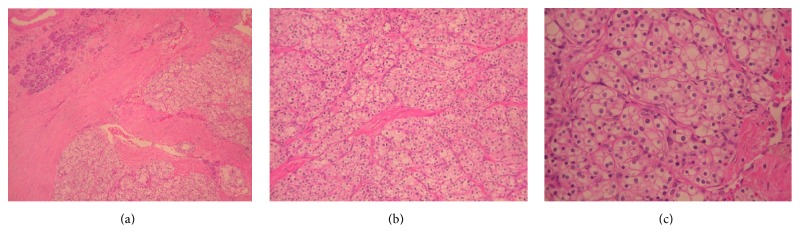
Histological examination. (a) The tumor shows well-defined borders with thick fibrous capsule (H&E ×100). (b) Clear cells in solid nests and few tubules (H&E ×200). (c) Clear cells in solid nests with round nuclei without atypia or mitoses (H&E ×400).

A 72-year-old woman presented to our clinic having nonspecific dyspeptic disturbances and mild pain in the epigastrium. She had no other symptoms and was free from any underlying disease. The patient had a medical history that included thyroidectomy (5 months earlier), tumor excision of her left breast (2 years earlier), and cholecystectomy and appendectomy (both more than 10 years earlier). The patient had an unremarkable family history and was not taking any medications. Other pertinent details from her medical history were that she was allergic to an unknown antibiotic and she did not smoke or drink alcohol. Physical examination revealed mild abdominal tenderness at the epigastrium and left hypochondrium and mild tenderness at her left breast, but no other signs. The patient was completely fit, and her vital signs and laboratory examinations (including tumor marker tests) were all within normal ranges. The patient underwent magnetic resonance imaging (MRI) which revealed a 3 cm, well-demarcated, enhanced mass in the tail of the pancreas (Figures 1(a) and 2(b)).

She had no other imaging exams, and she was scheduled for surgery with the suspicion that the tumor was a nonfunctional pancreatic neuroendocrine tumor (NET). Intraoperatively, a well-encapsulated mass was recognized at the tail of the pancreas and distal pancreatectomy without splenectomy was performed by laparotomy. The patient's recovery was complicated by a pancreatic fistula that lasted for ten days and resolved spontaneously with conservative treatment. The patient had no other events, and she was discharged from the hospital on the fifth postoperative day.

Histological examination demonstrated a solid serous adenoma of the pancreas. Gross examination showed a well-demarcated tumor. Microscopic examination of the neoplasm showed a tumor with well-defined borders with a fibrous capsule (Figure 2(a)) that consisted of clear cells located in solid nests with a few tubules (Figure 2(b)). The solid nests had round nuclei and were without atypia, mitoses, or necrosis (Figure 2(c)).

**Figure 3:**
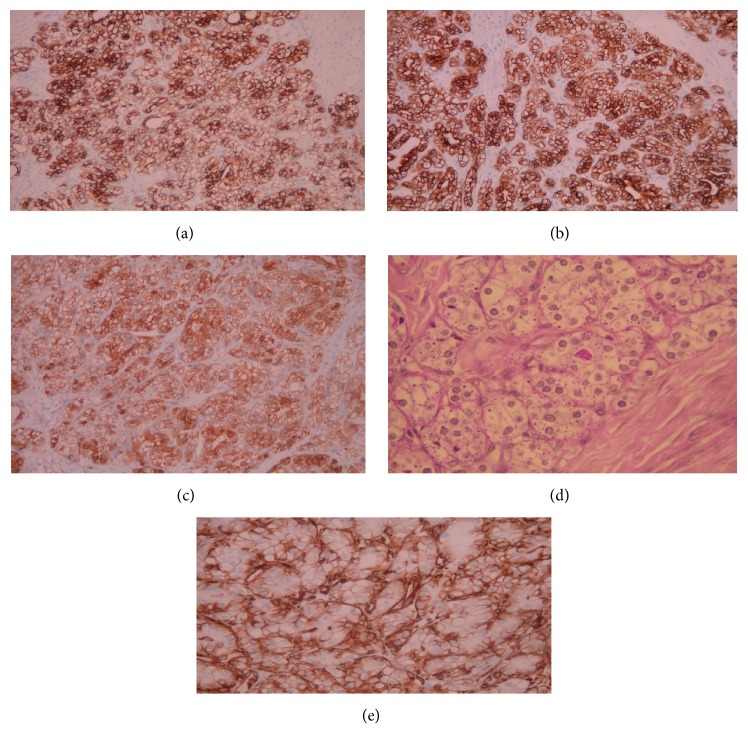
Immunohistochemical examination. (a) The tumor cells are positive for Cytokeratin-7 (Immunostain ×200). (b) The tumor cells are positive for Cytokeratin-8/Cytokeratin-18 (Immunostain ×200). (c) The tumor cells are positive for NSE (Immunostain ×200). (d) Intracytoplasmic PAS positive granules (PAS ×400). (e) Focal basolateral membranous staining for vimentin (Immunostain ×400).

The adjacent pancreatic tissue was completely normal, and invasion of the capsule was not discovered in any tumor specimens. Immunohistochemical examination revealed cells positive for each of the following: Cytokeratin-7 (Figure 3(a)), Cytokeratin-8/Cytokeratin-18 (Figure 3(b)), neurospecific enolase (NSE) (Figure 3(c)), and intracytoplasmic periodic acid-Schiff (PAS) (Figure 3(d)). All cells were negative for vimentin (Figure 3(e)).

Considering all these findings, the tumor was diagnosed as a solid serous adenoma of the pancreas. The patient was followed up one month and six months postoperatively, and we observed that she has recovered completely and remains disease-free.

## 3. Discussion

Cystic neoplasms of the pancreas are a type of tumor that appears rarely and generally has a good prognosis [1–4]. Diagnosing a cystic neoplasm is difficult preoperatively, and it is usually misdiagnosed as an NET [1–3]. Cystic neoplasms represent only 1-2% of pancreatic tumors, and they are important due to their biological behavior [4, 8].

Serous cystic neoplasms of the pancreas are subdivided into the five types mentioned previously, and the SSC is the rarest of those, with only 15 cases having been reported worldwide [1, 2]. The first described SSC was reported in 1996 by Perez-Ordonez et al., and since then only 14 more cases had been reported before the case reported herein, which, to the authors' knowledge, is the first SSC ever reported in Greece [4, 5, 8]. It is important for surgeons and radiologists to be aware of this entity, because its images resemble those of other solid tumors, including renal cell metastasis, NETs, and solid pseudopapillary tumors [1, 4].

The SSC type of cystic tumor generally is small (about 3 cm, ranging in size from 2,5 to 4,0 cm), has no sex predominance, and usually develops in the seventh decade of life [1–3]. Clinical diagnosis of an SSC cystic tumor is difficult because it cannot be distinguished from other solid tumors due to its radiologic characteristics which are similar to those of a solid tumor and which do not distinguish it as a cystic tumor [1, 4–7, 9–11]. Radiologic images such as those of CT and magnetic resonance imaging (MRI) are not diagnostic, and even endoscopic ultrasound-fine needle aspiration (EUS-FNA) can fail to support the diagnosis of an SSC neoplasm, even though it can support the diagnosis of an NET tumor when necrosis is found in the specimen on histopathological examination [1, 2, 4, 12]. So far, there are only two reported cases in which an SSC was correctly diagnosed preoperatively [1, 10, 11, 13]. Based on clinical imaging, most of the other cases were thought to be NET tumors [1, 2, 10, 11, 13]. Data reported for SSC tumors show that they present with strong enhancement in the early phase of a dynamic CT scan and with weak enhancement in their centers during the early-to-late phase [1, 4]. This phenomenon is explained by their high vascularity and rapid washout rate [1, 9]. Another explanation for this image in the CT scan may be the rich vascularity that appears at the margins of these tumors and their surrounding fibrous capsules [1, 9]. MRI images from published reports of previous tumors point to the fact that SSC tumors show a marked high intensity on T2-weighted images, higher than that of NET tumors [1, 2, 4, 10, 12]. An EUS-FNA is not adequate to support the diagnosis of an SSC tumor, because it cannot provide samples to support the diagnosis, even though it is a useful examination for other solid tumors of the pancreas [1].

SSC neoplasms of the pancreas grow slowly and do not have malignant predispositions [1, 2, 4, 8]. If there is high suspicion that a pancreatic tumor is an SSC neoplasm, based on radiological images such as CT and magnetic resonance imaging-magnetic resonance cholangiopancreatography (MRI-MRCP) and an EUS-FNA that is negative for malignancy, surgery can be avoided or minimized to more conservative operations, and close follow-up is all that is needed [1, 2, 4].

SSC neoplasms of the pancreas present difficulties in preoperative diagnosis and special immunohistochemical characteristics [4, 8]. They are usually discovered incidentally or during exams for mild, nonspecific pain in the epigastrium [1, 2]. They can be located anywhere in the pancreas (head, neck, body, or tail), and their mean size is about 3 cm [3, 4]. They are more common in women and people in the seventh decade of life [4, 14]. Most patients who have an SSC tumor undergo a CT scan, and the tumor's mass is observed as a well-defined, enhanced, solid tumor of the pancreas [1, 4]. Most of the cases that have been reported to date were first misdiagnosed as NETs or undefined tumors of the pancreas [1, 4]. Radiologically, an SSC adenoma is hypoechoic on ultrasound and shows contrast enhancement on CT, MRI, and arteriography [1, 2, 4]. An interesting characteristic of this adenoma is that it does not invade or compromise surrounding tissues, and, as a result, the common bile duct and the main pancreatic duct are never dilated [2]. In fact, dilation of the bile duct or the pancreatic duct must lead to suspicion of a malignant tumor rather than a benign tumor like an SSC adenoma [4]. As has been demonstrated by some authors [10, 12], an SSC adenoma of the pancreas can be diagnosed preoperatively by MRI T2-weighted imaging, and some of its characteristics, including well-defined borders and intense contrast enhancement on CT and MRI, can differentiate it from adenocarcinomas or intraductal papillary mucinous neoplasm (IPMN) tumors [2, 4]. If it cannot be distinguished or if malignancy is suspected, surgical excision is the treatment of choice, and it can be achieved by enucleation or pancreatic resection [2–4, 8]. The prognosis is good, and, in all cases that have been reported to date, the patients are alive with no reported recurrence [1–4, 8].

Pathological findings reveal that SSC adenomas are made of serous cystadenoma cells that lack the capability to secrete fluid and as a result of this fact they have lost the typical architecture of a serous cystadenoma [2, 8]. Gross examination shows a well-defined, solid mass with thick, fibrous bands at its cut surface, absence of necrosis or hemorrhage, and lack of invasion of adjacent tissues [2, 4]. Microscopic examination reveals glandular spaces; well-demarcated margins; solid, irregular nests at the stroma; rich, vascular fibrocollagenous tissue at the center; and thick, hypocellular fibrous bands at the surface of the tumor or SSC adenoma [2, 4, 8]. Immunohistochemical analysis of SSC adenomas of the pancreas develops positivity for Cam 5.2, CK7, PAS, epithelial membrane antigen (EMA), NSE, alpha-inhibin, MUC6, and calponin [2, 4, 8]. In some cases, antibodies such as Lu5, MUC1, AE1/AE3, Ca19-9, and MA902 may also be positive [2]. Staining for other antibodies, including synaptophysin, chromogranin, insulin, glucagons, gastrin, somatostatin, vasoactive intestinal peptide (VIP), serotonin, lipase, calcitonin, trypsin, and chromotrypsin, is usually negative [2, 4, 8].

An SSC adenoma of the pancreas is composed of cells with clear cytoplasm and must be differentiated from other tumors with clear-cytoplasm cells, such as ductal adenocarcinoma, solid pseudopapillary neoplasm, primary clear-cell sugar tumor or perivascular epithelioid cell tumors (PEComa), endocrine tumors of the pancreas (clear-cell islet tumors), and metastatic, clear-cell renal cell carcinoma (RCC) of the pancreas [4, 8].

As the literature shows, SSC is a tumor type that has a good prognosis and no recurrence [1–4]. Diagnosis based on radiologic findings is difficult, and it is very important to exclude malignancy before any therapeutic intervention [1, 2, 4]. The architecture of an SSC adenoma is different from that of a serous cystadenoma, but the cytological, immunological, and histopathological characteristics of the two are identical [8]. Surgical excision offers a cure, but follow-up with periodic imaging is necessary [4, 8]. SCC is a very rare entity that surgeons and pathologists must recognize in order to offer the correct treatment [3, 4].
